# Enzyme replacement therapy in infants and very young children with Gaucher disease using velaglucerase alfa: a single-center experience

**DOI:** 10.3389/fped.2025.1613599

**Published:** 2025-10-17

**Authors:** Ozlem Goker-Alpan, Margarita M. Ivanova, Ravi Pathak, Ekaterina Wright

**Affiliations:** ^1^ Lysosomal & Rare Disorders Research & Treatment Center, Fairfax, VA, United States; ^2^ Takeda Pharmaceuticals USA, Inc., Lexington, MA, United States

**Keywords:** Gaucher disease, pediatrics, enzyme replacement therapy, velaglucerase alfa, hepatomegaly, splenomegaly, lyso-Gb1

## Abstract

**Objective:**

To evaluate the effectiveness and safety of enzyme replacement therapy (ERT) with velaglucerase alfa, and offer insights into the clinical course of patients with Gaucher disease (GD) that were diagnosed and treated early in life.

**Study design:**

A phase IV, observational, retrospective and prospective study (NCT04721366) enrolled children with GD who initiated velaglucerase alfa under 4 years of age. Of twelve patients screened, 11 were enrolled (six boys, five girls; two retrospectively); four were identified through newborn screening (NBS).

**Results:**

Mean age of diagnosis was 14 months (range, 2 weeks–38 months) and most patients presented with splenomegaly. Patient genotypes included glucosylceramidase beta 1 gene variants R163X, L444P, R463C, N462K, D409H, 55-bp deletion, and other recombinant alleles. Velaglucerase alfa (60–80 U/kg) was initiated at age ≤3 months (*n* = 4), >3–≤6 months (*n* = 2), >6–≤12 months (*n* = 1), >12–≤18 months (*n* = 2), and >36–≤48 months (*n* = 2), administered weekly/every other week, mostly in home settings. Most patients were treated for ≥12 months (range, 2–57 months). Hematological values, organ sizes, and growth parameters improved and/or remained stable for all patients; no typical GD-related bone manifestations were observed. Glucosylsphingosine levels decreased from 90–874 ng/mL to 4–26 ng/mL within 6 months of starting ERT. No drug-related adverse events were recorded.

**Conclusions:**

These preliminary data suggest that velaglucerase alfa is well-tolerated and associated with improvements in clinical parameters in very young children with GD types 1 and 3, offering insights into the early presentation and course of GD in infancy and early childhood.

## 1 Introduction

Gaucher disease (GD) is caused by pathogenic variants in the glucosylceramidase beta 1 gene (*GBA1,* NM_000157.4*)* (1, 2), leading to deficient activity of the lysosomal enzyme acid β-glucocerebrosidase and an accumulation of glucosylceramide and other sphingolipids in the tissues and organs of the reticuloendothelial system (1, 3). To date, more than 500 pathogenic variants of *GBA1* have been described, including frame-shift, point, or splice site mutations, and deletions, insertions, or recombinant alleles (4). GD is a multi-system disorder characterized by phenotypic heterogeneity and a wide clinical spectrum (5). Common presentations include hepatosplenomegaly, thrombocytopenia, anemia, delayed growth, and bone disease (1, 6). Distinction among different phenotypes helps the clinician to not only predict the disease course, but also provide guidance to patients.

Clinical classification of GD is determined according to the primary involvement and progression of central nervous system manifestations (6). GD type 1 (GD1; OMIM 230800) is defined as the non-neuronopathic form, which accounts for 94% of GD cases in the Western hemisphere; it can present at any age and is clinically heterogenous (2, 3). Neuronopathic GD includes GD type 2 (GD2; OMIM 230900) and GD type 3 (GD3; OMIM 231000), and is pan-ethnic, accounts for the majority of cases in the rest of the world, and has an estimated birth incidence of 1 in 100 000–300 000 (3). GD2 represents the acute neuronopathic subtype and it can manifest from the perinatal or neonatal period to early infancy; it is associated with brain stem involvement and is invariably fatal in early childhood (2, 3). GD3 is the subacute neuronopathic subtype; this often has a clinical onset in early childhood and is characterized by the universal finding of slowed horizontal saccades (2, 3). Clinical presentation of patients with GD3 is heterogenous, and is characterized by severe systemic involvement including enlarged liver and spleen, anemia, thrombocytopenia, bone manifestations with progressive kyphosis and kyphoscoliosis, infiltrative lung disease, and neurological involvement including seizures, ataxia, dystonia, and various degrees of cognitive impairment (6, 7). While a typical neurological finding in GD3 is impaired saccadic eye movements, phenotypic heterogeneity has resulted in further subclassifications for GD3. The subtype GD3a is associated with myoclonic epilepsy, and occasionally manifests with neurological disease that may precede systemic presentation. Subtype GD3b is the most common subtype and is characterized by extensive visceral involvement. Subtype GD3c is associated with the genotype D448H (D409H)/D448H (D409H), presenting with cardiac calcifications, corneal opacities, and occasionally hydrocephalus, and with the genotype L483P (L444P), also referred to as the Norbottnian subtype; patients who are homozygous for the L483P allele have a phenotype with progressive kyphosis and early onset visceral and neurological manifestations with progressive cognitive decline (6, 8–10).

Visceral manifestations are present in all types of GD and include splenomegaly, hepatosplenomegaly, thrombocytopenia, anemia, bone marrow infiltration and failure, Erlenmeyer flask deformities, osteopenia, osteonecrosis, pathological fractures, and delayed growth or puberty (1, 8, 11).

Children with GD are usually evaluated because they present with thrombocytopenia, anemia, and/or organ enlargement (splenomegaly or hepatosplenomegaly) (2, 6). In a recent study conducted among children with splenomegaly, thrombocytopenia, and/or anemia, the prevalence of GD was found to be significantly higher than in similarly affected adults. In the same cohort, the pediatric patients with GD also had significantly lower hemoglobin levels, higher ferritin levels, and higher rates of growth delay than other children with splenomegaly but without GD (12).

Many patients with GD are misdiagnosed or remain without a conclusive diagnosis for many years until irreversible complications ensue (13). Large-scale screening efforts aim to not only avoid misdiagnosis, but also allow early diagnosis and treatment, which are critical to prevent serious and irreversible complications such as skeletal deformities and chronic pain, and to improve outcomes and quality of life for patients. With the introduction of newborn screening (NBS) for GD, practiced in a few states in the USA (2, 6), it is essential to develop guidelines for the clinical management and treatment of symptomatic infants and very young children with GD.

Enzyme replacement therapy (ERT) is the standard of care, and is recommended for all symptomatic children or adolescents with GD (1, 6). Velaglucerase alfa is used for the long-term treatment of the systemic manifestations of GD in adult and pediatric patients aged 4 years and older (14). However, there is limited evidence on velaglucerase alfa in children aged younger than 4 years. This report presents preliminary real-world evidence on the use of velaglucerase alfa as ERT in infants and very young children with GD, exploring its potential effectiveness and safety in this understudied age group. In addition, we provide further information on the clinical course of patients with GD that was diagnosed and treated early in life.

## 2 Subjects and methods

### 2.1 Study design and patient selection

This was a combined retrospective and prospective observational study (clinicaltrials.gov: NCT04721366) conducted at the Lysosomal & Rare Disorders Research and Treatment Center in Fairfax, VA, USA, which enrolled patients with GD who initiated treatment with velaglucerase alfa before the age of 4 years. The protocol was approved by the local institutional review board and the study was conducted in compliance with the International Conference on Harmonisation Good Clinical Practice guidelines and the Declaration of Helsinki. For all patients, written informed consent was obtained from the parent(s) or legally authorized guardian(s). Patients were enrolled between January 8, 2021, and December 13, 2022.

Patients with currently diagnosed GD1 or GD3 that was confirmed biochemically (enzymatic activity of leukocyte β-glucosidase) and verified genetically (*GBA1* genotyping) who initiated ERT before the age of 4 years were eligible. Data were compiled about clinical practices regarding the start, dose, and frequency of ERT, and clinical parameters such as hematologic values, bone and growth parameters (weight and height centiles, and bone age), and liver and spleen sizes when available. Clinical information was also gathered regarding the neurological and neurocognitive status. Data were collected retrospectively for up to 36 months and/or prospectively for up to 18 months, for a total treatment period of up to 36 months. Patients were treated in accordance with the physician's treatment plan per standard clinical practice with a prescribed velaglucerase alfa dose of 60 U/kg every other week (EOW).

The number of drug-related adverse events was a secondary endpoint. Adverse events were recorded using parent statements and/or chart review.

### 2.2 Clinical and laboratory assessments

Hemoglobin levels, platelet counts, liver and spleen sizes, growth normalization, bone age and bone density, as well as improvements in organomegaly and thrombocytopenia were assessed from velaglucerase alfa initiation up to 5 years of age. The earliest available measurement was defined as study enrollment or ERT initiation, whichever was earlier. Liver and spleen sizes were assessed using ultrasonography, and organ lengths were provided in centimeters and compared with the normal measurements for age (15, 16). Bone involvement was assessed through skeletal surveys (x-rays) to evaluate for GD-related abnormalities, such as Erlenmeyer flask deformities, osteopenia, or lytic lesions. Bone age was determined using x-rays of the wrist or knee, compared to chronological age using standard reference charts. Pediatric Whole Body Dual-Energy X-ray Absorptiometry (DEXA) (excluding the head) was used to assess bone mineral density where applicable.

**Determination of enzymatic activities and GD biomarkers:** Data on GD-specific biomarkers, including glucosylsphingosine (Lyso-Gb1) and chitotriosidase, were also recorded. Lyso-Gb1 was measured commercially (LabCorp) in whole blood samples. Glucocerebrosidase (GCase) and chitotriosidase activity were assessed using commercially available kits in samples obtained from dried blood spots (DBSs) as previously described (17).

Data for the earliest available measurements were entered from historical records (subject to data availability) for patients who had already initiated treatment with velaglucerase alfa.

**Chitotriosidase enzymatic analysis:** Chitotriosidase activity in DBS samples was determined using the fluorogenic substrates that can be hydrolyzed by chitinase, 4-methylumbelliferyl-β-D-N,N′,N″-triacetyl-chitotrioside (4MU-C3, Sigma). The assay was performed according to the Hollak method (18). The reaction was incubated for 3 hours and then stopped using glycine-sodium hydroxide buffer. The signal was measured with an emission wavelength of 360 nm and an excitation wavelength of 450 nm using the SpectraMax M2 instrument from Molecular Devices. The activity of the enzyme β-glucocerebrosidase was measured using the fluorometric substrate 4-methylumbelliferyl β-D-glucopyranoside, following the original method developed by Chamoles et al. (19). Briefly, two 1 mm punches from DBS were extracted using a 0.1 M citrate-phosphate buffer (pH 5.6) and incubated for 1 hour. After extraction, the assay was conducted using 0.1 M citrated buffer, pH 5.2, supplemented with sodium taurocholate (0.8% w/v) for 20 hours at 37°C, and then stopped using a 0.2 M glycine sodium hydroxide buffer (pH 10.7) (20). Fluorescence was measured at an excitation wavelength of 355 nm and an emission wavelength of 460 nm using a FilterMax F5 (Molecular Devices). The enzyme activities were expressed as nanomoles of hydrolyzed substrate per hour per milliliter of blood.

### 2.3 Statistical analysis

This was a feasibility study in which no sample size estimation was performed. Twenty patients were planned to be included; however, the study was closed early owing to challenges with enrollment, in part due to the coronavirus 2019 (COVID-19) pandemic. Statistical evaluation was descriptive, with no hypotheses tested and without imputation of missing values.

## 3 Results

### 3.1 Patient characteristics

Twelve patients were screened, and eleven (six boys and five girls) were enrolled (Table 1). Two patients were enrolled in the retrospective arm of the study, and nine were followed prospectively, with previously available data also collected. Only 7 of the 11 patients remained in the study for ≥20 months, and one patient received therapy for 6 weeks. Three patients (F, H, J) discontinued the study: two enrolled in an interventional trial and one developed neurological symptoms consistent with GD2, which precluded their ongoing study participation. While clinical data were presented here, only safety data from the patients who discontinued the study were included in the actual analysis.

**Table 1:** Patient demographics and clinical characteristics.

Characteristic	Patient ID										
	A	B	C	D	E	F	G	H	I	J	K
Study participation	Completed	Ongoing at study closure	Completed	Ongoing at study closure	Ongoing at study closure	Discontinued	Completed	Discontinued	Completed	Discontinued	Ongoing at study closure
Enrollment type	Prospective	Prospective	Prospective	Prospective	Prospective	Prospective	Retrospective	Prospective	Retrospective	Prospective	Prospective
Sex	Female	Female	Male	Male	Male	Female	Male	Female	Male	Male	Female
GD clinical phenotype	3	3	1/3	1/3	3c	2	2/3	2	3	2	2/3
*GBA1* genotype											
Allele 1	c.604C > T p.Arg202Ter	c.1448T > C p.Leu483Arg	c.1448T > C p.Leu483Arg	c.1448T > C p.Leu483Arg	c.1342G > C p.Asp448His	c.1342G > C p.Asp448His	c.1448T > C p.Leu483Arg	c.1265_1319del p.Leu422fs Deletion, 55 bp (frameshift)	c.1448T > C p.Leu483Arg	c.203del Deletion	c.1342G > C p.Asp448His (D409H)
Allele 2	c.1246G > A p.Gly416Ser	c.1503C > G p.Asn501Lys	c.1504C > T p.Arg502Cys	c.1448T > C p.Leu483Arg	c.1342G > C p.Asp448His	c.203dup p.Thr69fs (frameshift)	Recombinant^a^	c.1448T > C p.Leu483Arg	c.1448T > C p.Leu483Arg	c.1448T > C p.Leu483Arg	Rec*NciI*
GCase activity at diagnosis, nmol/h/mL	0.5	0.17	N/A	101	0.00674	1	0.416^b^	0.00214	1.3	0.00031	0
Age at diagnosis, months^c^	>36–≤50	>1–≤3	>24–≤36	≤1 (including NBS)	>6–≤9	>1–≤3	>9–≤12	≤1 (including NBS)	>12–≤18	≤1 (including NBS)	≤1 (including NBS)
Lyso-Gb1 levels at presentation, ng/mL (Normal level: < 0.05 ng/mL)	238 at >36–≤50 months	141 at ≤2 months	100 at >9–≤12 months	162 at >9–≤12 months	115 at >9–≤12 months	N/A	874 at >9–≤12 months	N/A	N/A	90 at ≤2 months	183 at >1–≤3 months
Chitotriosidase activity at presentation, nmol/h/mL (Normal mean level: 20 nmol/h/mL; upper limit: 120 nmol/h/mL)	11 459 at >36–≤50 months	650 at ≤2 months	–^d^	413 at >9–≤12 months	768 at >9–≤12 months	370 at ≤2 months	–^d^	1,733 at >1–≤3 months	6,477 at >12–≤18 months	583 at ≤2 months	677 at >1–≤3 months
Weight percentile (age in months)	90%ile (39)	45%ile (9)	77%ile (42)	45%ile (19)	<3%ile (33)	13%ile (3)	16%ile (49)	<2%ile (3)	56%ile (48)	60%ile (2)	30%ile (3)
Height percentile (age in months)	27%ile (39)	62%ile (9)	20%ile (42)	60%ile (19)	<3%ile (33)	19%ile (3)	2%ile (49)	<2%ile (3)	38%ile (48)	44%ile (3)	60%ile (3)
Head circumference percentile (age in months)	N/A^e^	86%ile (9)	94%ile (40)	25%ile (19)	7%ile (19)	24%ile (3)	25%ile (49)	10%ile (2)	N/A^e^	10%ile (2)	17%ile (3)

*GBA1*, glucosylceramidase beta 1 gene; GCase, glucocerebrosidase; GD, Gaucher disease; Lyso-Gb1, glucosylsphingosine; N/A, not available; NBS, newborn screening; SD, standard deviation.

^a^ Recombinant allele (several pseudogene derivative variants located in exon 9 and 10).

^b^ Value taken closest to diagnosis.

^c^ Age information is given in ranges to protect patient anonymity.

^d^ Attributed to variants in the *Chitotriosidase 1* gene.

^e^ The growth percentiles or the numerical values for the growth parameters were not available.

The demographics, clinical types, and genotypes are listed in Table 1. Mean age at GD diagnosis was 14 months (range, 2 weeks–38 months); four patients were identified through NBS. The majority of children with GD presented with splenomegaly, two had spleen sizes at the upper limit of normal for age (Table 2) (16). *GBA1* genotypes were varied, including R163X, L444P, R463C, N462K, D409H, and 55-bp deletion, and other recombinant alleles (Table 1).

**Table 2:** Treatment patterns and clinical outcomes.

Clinical characteristic	Patient ID										
	A	B	C	D	E	F	G	H	I	J	K
Treatment											
Age at treatment initiation,^a^ months	>36–≤50	>1–≤3	>36–50	>3–≤6	>12–≤18	>1–≤3	>6–≤12	>3–≤6	>12–≤18	>1–≤3	>3–≤6
Treatment duration, months	20	27	20	32	20	18	54	12	57	9	1
IV access	Mediport	Mediport	Mediport	Central line/Mediport	Central line	PICC line/Mediport	Mediport	Mediport	Central line/Mediport	Mediport	Central line
Treatment location	Home	Home	Home	Home	Hospital	Home	Home	Home	Home	Home	Home
Dosage, U/kg	60 EOW	60 weekly	60 weekly	76.9 weekly	59 EOW	60 weekly	66 weekly	71 EOW	61 weekly	60 weekly	71 weekly
Clinical outcomes											
Hemoglobin level^b^ at earliest measurement, g/dL	11.9 (before treatment)	11.2 (before treatment)	12.8 (before treatment)	12.4 (before treatment)	13.4 (after treatment)	11.5 (after treatment)	8.8 (before treatment)	11.0 (after treatment)	11.9 (after treatment)	9.7 (before treatment)	11.4 (before treatment)
Hemoglobin level at the latest measurement, g/dL	12.6 (after treatment)	12.0 (after treatment)	12.3 (after treatment)	11.6 (after treatment)	11.5 (after treatment)	N/A	11.9 (after treatment)	N/A	13.0 (after treatment)	N/A	N/A
Platelet count^b^ at the earliest measurement, thousand/*μ*L	132 (before treatment)	113 (before treatment)	129 (before treatment)	312 (before treatment)	179 (after treatment)	324 (after treatment)	151 (before treatment)	217 (after treatment)	207 (after treatment)	281 (before treatment)	220 (before treatment)
Platelet count^b^ at the latest measurement, thousand/μL	202 (after treatment)	319 (after treatment)	229 (after treatment)	318 (after treatment)	109 (after treatment)	N/A	217 (after treatment)	N/A	207 (after treatment)	N/A	N/A
Liver size (cm)											
Liver size at presentation^c^, cm (time before, after, or at treatment initiation)	11.4 (2 months before treatment initiation)	8.2 (1 month before treatment initiation)	Normal (31 months before treatment initiation)	7.8 (1 month before treatment initiation)	8.6 (6 months before treatment initiation)	Normal (1 month after treatment initiation)	10.5 (5 months after treatment initiation)	5.9 (3 months before treatment initiation)	11.0 (at treatment initiation)	9.5 (at treatment initiation)	10.0 (0.5 months before treatment initiation)
Intermediate measurements, cm (time after treatment initiation)	11.1 (5 months)	N/A	Mildly enlarged (10 months)	N/A	N/A	N/A	9.8 (15 months)	N/A	10.5 (10 months) 11.5 (32 months)	N/A	N/A
Latest measurement, cm (time after treatment initiation)	9.5 (14 months)	9.0 (17 months)	12.1 (12 months)	298 mL (21 months)	Normal (29 months)	N/A	9.39 (39 months)	7.6 (5 months)	Normal (58 months)	Normal (2 months)	N/A
Spleen size (cm)											
Spleen size at presentation,^d^ cm (time before, after, or at treatment initiation)	10.5 (2 months before treatment initiation)	7.5 (at treatment initiation)	14.0 (31 months before treatment initiation)	7.0 (1 month before treatment initiation)	10.6 (6 months before treatment initiation)	7.4 (1 month after treatment initiation)	Normal (5 months after treatment initiation)	7.4 (3 months before treatment initiation)	15–16 (at treatment initiation)	6.2 (at treatment initiation)	7.1 (0.5 months before treatment initiation)
Intermediate measurements, cm (time after treatment initiation)	7.8 (5 months)	N/A	12.1 (10 months)	N/A	N/A	N/A	7.8 (15 months)	N/A	9.1 (10 months) 9.6 (32 months)	N/A	N/A
Latest measurements, cm (time after treatment initiation)	8.4 (14 months)	7.9 (17 months)	13.6 (12 months)	7.1 (33 months)	9.0 (29 months)	N/A	7.6 (39 months)	Normal (5 months)	Normal (58 months)	N/A	N/A

EOW, every other week; IV, intravenous; N/A, not available; PICC, peripherally inserted central catheter.

^a^ Age information is given in ranges to protect patient anonymity.

^b^ Age specific hematological values from Forestier F et al. Pediatr Res 1986, Oski FA, Naiman JL WB Saunders 1982.

^c^ According to Waelti *et al. BMC Pediatrics* 2021:21;276 (15), 5th–95th percentiles for right liver lobe craniocaudal diameter were 5.2–8.3 cm in children aged >1–≤6 months, 5.4–10.0 cm in children in aged >6–≤12 months, 6.2–10.3 cm in children aged >1–≤2 years, 7.1–10.8 cm in children aged >2–≤3 years, 8.3–11.0 cm in children aged >3–≤4 years, 8.8–11.2 cm in children aged >4–≤5 years, and 8.9–12.1 cm in children aged >5–≤6 years.

^d^ According to Rosenberg et al. *AJR Am J Roentgenol* 1991:157(1):119–21 (16), 10th–90th percentiles for spleen length were 3.3–5.8 cm in children aged >0–≤3 months, 4.9–6.4 cm in children aged >3–≤6 months, 5.2–6.8 cm in children in aged >6–≤12 months, 5.4–7.5 cm in children aged >1–≤2 years, 6.4–8.6 cm in children aged >2–≤4 years, 6.9–8.8 cm in children aged >4–≤6 years, and 7.0–9.6 cm in children aged >4–≤6 years.

### 3.2 Treatment patterns

The initiation of ERT was determined based on clinical symptoms and elevated Gaucher disease-specific biomarkers. Velaglucerase alfa was started at the following ages: ≤3 months in four patients, >3–≤6 months in two patients, >6–≤12 months in one patient, >12–≤18 months in two patients, and >36–≤48 months in two patients. Patients received treatment for up to 57 months, with nine patients treated for 12 months or longer. Of eight patients who remained in the study, seven were treated with velaglucerase alfa for 20 months or more. The initial prescribed dosage of velaglucerase alfa was 60 U/kg EOW as per the clinical management plan; however, patients received doses of 59–77 U/kg according to clinic practice and individual clinical needs. The frequency of velaglucerase alfa administration was EOW in three patients and weekly in eight patients. One patient started receiving weekly infusions then continued with an EOW schedule (Table 2). Velaglucerase alfa was administered using central venous access, usually starting with a central line and switching to a mediport; all patients received home infusions except for one (treated at an infusion center because of parental preference). The youngest age for port placement was 2 months.

### 3.3 Clinical outcomes

Initial hemoglobin levels and platelet counts ranged from 8.8 g/dL to 13.4 g/dL and from 113 × 10^9^/L to 324 × 10^9^/L, respectively (Table 2). Hematological parameters improved and/or remained stable after velaglucerase alfa initiation for all patients with available measurements for pre- and post-treatment initiation (*n* = 5), and were maintained throughout the study. At the latest recorded measurement during treatment, for those patients with available data (*n* = 7), hemoglobin levels were 11.5–13.0 g/dL and platelet counts were 109–319 × 10^9^/L (Table 2).

Liver size decreased in patients (A, G) and was within normal limits in seven patients at their latest measurement (Table 2). Spleen size decreased relative to normal values in all seven patients who remained in the study and were treated with velaglucerase alfa for 6 months or longer (five of whom received weekly velaglucerase alfa). Spleen size normalized for age in all but one patient (C) who had some residual splenomegaly despite a decrease in spleen size.

Head circumference percentiles ranged from 94% to 10%, and height percentiles ranged from 60% to 1% (Table 1) before velaglucerase alfa treatment. Weight percentiles for age ranged from 90% to 1%. Failure to thrive was seen in two patients: one patient with GD2 who discontinued the study and another whose weight increased from the first to the fifth percentile during treatment (this patient's growth followed normal growth curves and was within the predicted mid-parental heights).

A skeletal survey was available for all but one patient before treatment initiation: none had any bone abnormalities, including congenital deformities, Erlenmeyer flask deformities, or cystic or lytic lesions. There were no events associated with GD-related bone involvement throughout follow-up. Skeletal x-rays, taken in four patients, showed bone age consistent with chronological age. On follow-up radiological evaluation, one patient developed mild metaphyseal broadening at the long bones (patient A); bone density abnormalities of the radius were noted for another patient whose bone density was at the 2.7-percentile for age (patient E).

Although not prespecified in the clinical trial protocol, neurological assessments were documented when available. Two patients had normal eye movements, thus one was considered to have GD1 (patient C), while the other patient had a genotype consistent with GD3 (L483P/L483P). All other patients had horizontal supranuclear palsy, and one also had strabismus. Hearing abnormalities were the most common neurological finding, and were diagnosed in six patients with an abnormal auditory brainstem response at presentation. One patient received cochlear implants. Gait abnormalities including ataxia and spasticity were observed in three patients, among whom one was non-ambulatory. One patient had an abnormal electroencephalogram, myoclonus, and generalized seizures. All other individuals did not have clinical seizures and had normal ‘sleep deprived’ electroencephalograms.

### 3.4 GD biomarkers

**Figure 1:**
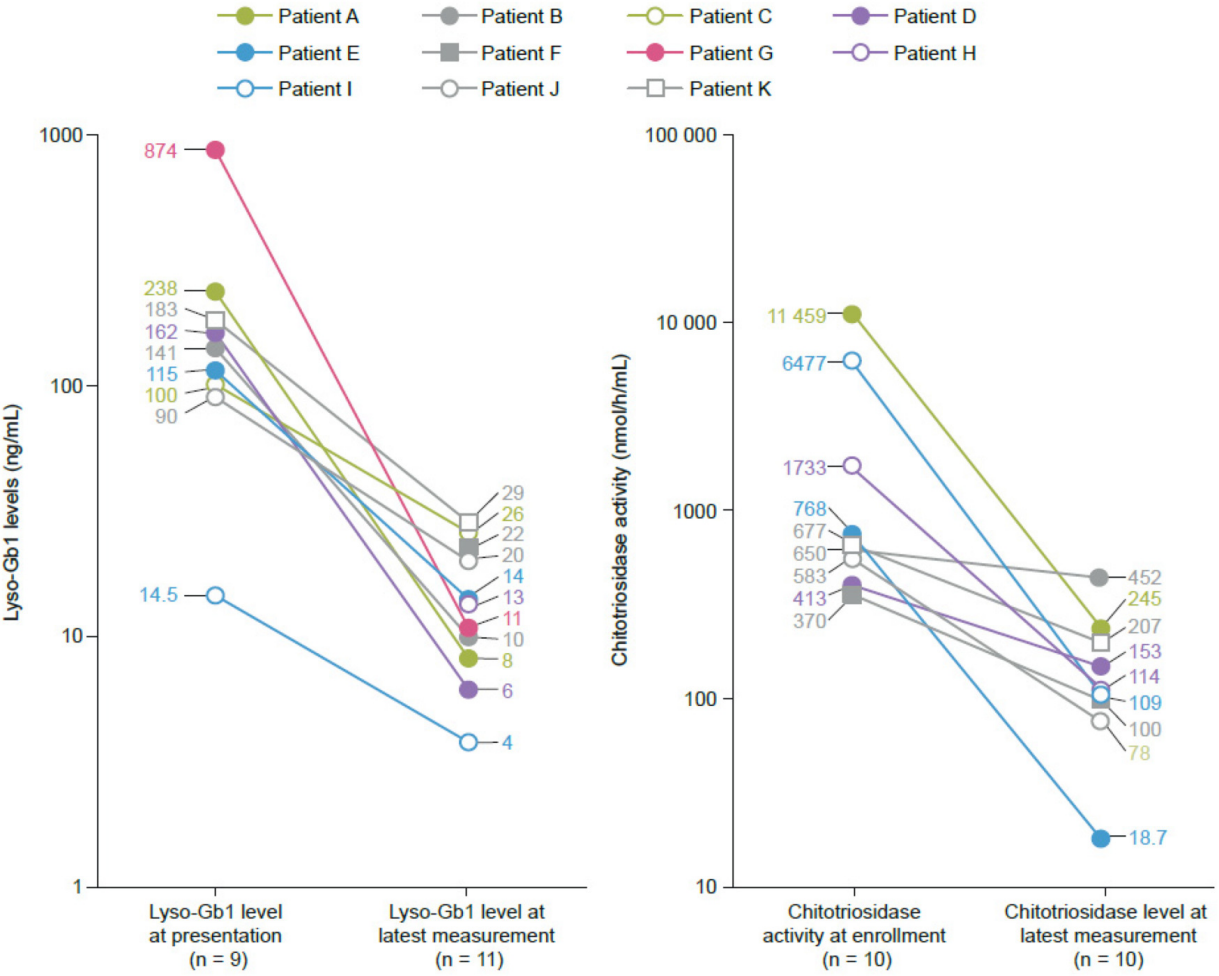
Reductions in glucosylsphingosine (lyso-Gb1) and chitotriosidase activity from baseline to the latest measurement in seven patients who remained in the study. Age at latest measurement (months): >2–≤6 (Patient B), >12–≤18 (Patients C, D), >18–≤36 (Patient E), >36–≤50 (Patient A), >50–≤72 (Patient I). Normal ranges: Lyso-Gb1 (<0.05 ng/mL), chitotriosidase (mean 20 nmol/h/mL, upper limit 120 nmol/h/mL). Data points represent individual patient measurements, with ages provided in ranges to protect anonymity.

Lyso-Gb1 levels were 90–874 ng/mL at presentation in the eight patients with available measurements (normal values: <0.05 ng/mL). Chitotriosidase activities were 370–11 459 nmol/h/mL in nine patients (normal values: mean, 20 nmol/h/mL; upper limit, 120 nmol/h/mL). Two patients had chitotriosidase activities below the normal mean at presentation, attributed to variants in the chitotriosidase gene*,* and thus were not followed up further (21). There was a rapid decrease in Lyso-Gb1 levels from presentation values in the 6 months after starting therapy, with clinically acceptable values of 4–26 ng/mL at the latest measurement (Figure 1). For one patient (K), who only received velaglucerase alfa for 6 weeks before study closure, Lyso-Gb1 levels decreased from 183 ng/mL to 29 ng/mL. Chitotriosidase activity levels decreased in all nine patients and were below the upper limit of normal (120 nmol/h/mL) at the latest measurement in five patients (Figure 1). After an initial decrease, one patient (B) had an increase in chitotriosidase levels to 2,272 nmol/h/mL, attributed to a history of recent SARS-CoV2 infection, which was sustained for some weeks before starting to decrease again.

### 3.5 Safety

No drug-related adverse events or infusion-related reactions were observed. Premedications were not given before velaglucerase alfa infusions. Five patients had a SARS-CoV-2 infection; two were hospitalized but did not require ventilation, and three recovered from COVID-19 without any sequelee.

## 4 Discussion

Our study provides vital preliminary evidence on the effectiveness and safety of ERT with velaglucerase alfa in infants and children under 4 years old. This cohort, including infants and very young treated patients with GD, offers valuable insight not only into the early presentation/course of GD but also into effectiveness of early intervention and therapy. Of 11 patients initially treated, seven remained in the study and were treated with velaglucerase alfa for 20 months or longer. Overall, no drug-related adverse events or infusion-related reactions were observed, and all but one patient received home infusions.

ERT, as the standard of care, is currently the only treatment in the pediatric population approved by the European Medicines Agency and it is also offered in several states of the USA, despite not being approved for GD3 (6). Three phase 3 clinical trials and their extension studies have evaluated the safety and efficacy of velaglucerase alfa in GD, including children as young as 4 years old (22–24). A multinational, phase 3 trial evaluated two doses of velaglucerase alfa (60 U/kg or 45 U/kg) in 25 treatment-naive patients with symptomatic GD1 (of whom seven were aged between 4 years and 17 years), and demonstrated improvements in hemoglobin concentrations and platelet counts, and decreases in spleen and liver volumes (22). A phase 2/3, multicenter, open-label study demonstrated stable hemoglobin concentrations, platelet counts, and liver and spleen volumes in 40 patients who had previously received imiglucerase (23). Approximately a quarter of the patients in that study were younger than 18 years, the youngest being 9 years old (23). A third phase 3 study compared velaglucerase alfa with imiglucerase in a randomized, double-blind, noninferiority study of symptomatic treatment-naive patients with GD1 (24). It included four children less than 18 years of age treated with velaglucerase alfa (the youngest of whom was 7 years of age) and demonstrated similar effects with the two different ERTs on hemoglobin concentrations, platelet counts, and liver and spleen volumes (24).

Based on the findings from clinical studies, velaglucerase alfa was approved in the USA in 2010 for the long-term treatment of patients at least 4-years old with GD1 (14). The effects of velaglucerase alfa treatment in 24 pediatric patients who participated in a long-term extension study of the clinical studies leading to registration has been reported. The study found that the safety profile and clinical response seen in pediatric patients at least 4 years old were consistent with results reported in adults, and suggested benefits of early treatment to enable normal growth in pediatric patients (25). However, data on the effect of velaglucerase alfa treatment in children with GD younger than 4 years are lacking. A 6-year interim analysis of post-marketing surveillance in Japan reported that six patients under 4 years old had been treated with velaglucerase alfa (26). Efficacy was not reported separately for the pediatric subgroup, but adverse drug reactions, including pyrexia and vomiting, were observed in this Japanese population. No such reactions occurred in our US center, even in the absence of premedication (26).

The recommended initial velaglucerase alfa dosage is 60 U/kg EOW, for both adults and children (14). This recommended ERT dosing and frequency had been adopted from clinical trials that primarily included adults and older children with GD1. However, infants and young children presenting with GD symptoms early in life have higher disease loads and thus require more frequent and/or higher dose ERT regimens than recommended, similar to infantile Pompe disease (27–29). It is now recognized that the ERT dose should be individualized for each patient based on disease severity, rate of progression, and the achievement and maintenance of therapeutic goals (14, 30, 31). Such assessment should include comprehensive clinical, laboratory, and radiological evaluation, as well as consideration of the quality of life of the patient and the parents/caregivers. In our center, patients received velaglucerase alfa doses of 59–77 U/kg. Velaglucerase alfa was initially prescribed at 60 U/kg; however, because the dose was rounded up to the nearest unit dose per vial, there was an inadvertent increase from the prescribed dose. Thus, the actual dosing started from one vial (400 U) to three vials (1,200 U). This study demonstrates that the frequent (weekly) dosing schedule in infants and very young children was associated with a rapid decrease in biomarkers and improvement of organomegaly, hematological, and growth parameters for age. In addition, positive attitudes to weekly dosing were reported by the parents/caregivers.

Initiation of ERT immediately or as soon as possible after diagnosis is recommended for all symptomatic children with GD (6, 30, 32, 33). Studies of the effectiveness of ERT traditionally used clinical outcome measures that included, but were not limited to, hematological parameters, organ size, skeletal disease, lung involvement, and quality of life. However, traditional therapeutic goals fail to address long-term disease outcomes and associated complications such as Gaucheromas, lymphadenopathy, and infiltrative lung disease. Therefore, therapeutic goals were recently revisited to include long-term goals for pediatric patients with GD (6, 32–35). These include (skeletal) growth and development, normalization of growth, and normal pubertal development (6). The current therapeutic expectations for children still mostly emphasize improvement rather than attaining normalcy. Within 1–2 years after ERT treatment, targets include: increasing hemoglobin levels to over 11.0 g/dL, reducing and maintaining liver volume to within 1.0–1.5-times normal and spleen volume to below 2–8-times normal, improving bone mineral density and lessening or eliminating bone pain unrelated to irreversible bone disease, achieving normal height according to population standards and parental height within 2–3 years of treatment, and achieving normal onset of puberty (6).

The majority of patients with GD do well when ERT is administered early, before irreversible systemic and skeletal manifestations occur. As with any progressive condition, however, the stage of the disease at treatment initiation can have a significant effect on the clinical outcomes. A lack of timely diagnosis and/or therapy may lead to the development of irreversible bone complications in young adults or teenagers (36, 37). Similarly, treatment interruptions have been associated with adverse effects or long-term complications in both adults and children (6, 38).

In our cohort, the majority of patients had normalization of spleen and liver sizes after 6 months of treatment. Height percentiles remained generally stable or improved, and there was no evidence of GD-related bone involvement during up to 57 months of treatment in most patients. If therapeutic intervention occurs early enough before disease progression, even distinct signs of GD, such as Erlenmeyer flask deformities, can be alleviated. Although mild radiological changes have been observed in two patients (Patients A and E), early intervention will likely reduce the risk of severe GD-related bone complications for the future. Thus, early diagnosis through NBS and early intervention make long-term treatment goals possible, not only in terms of symptom improvement owing to effective management of complications, but also potential maintenance of life-long normalcy.

The biomarker Lyso-Gb1 is significantly elevated in pediatric patients with GD, indicating severe disease activity (39). Lyso-Gb1 is the deacylated form of the substrate glucosylceramide, a highly bioactive molecule primarily found in the lysosomes with the ability to diffuse through the membranes (40). Peripherally circulating Lyso-Gb1 is associated with cell damage and death, possibly induced by macrophages (41). Lyso-Gb1 levels correlate with other established markers of immune activation released from activated macrophages, such as chitotriosidase and CCL18 (39). Plasma chitotriosidase levels are also increased in children with GD, along with other markers of macrophage activation such as ferritin (12, 39). Macrophage activation is not only a marker of disease activity in GD, but also leads to the release of chemokines and cytokines downstream in the inflammatory pathway, thus contributing to further disease progression associated with immune dysregulation and dysfunction. Adults with a delayed diagnosis and/or delayed treatment of GD have irreversible immune perturbations despite any GD-specific treatment given in adulthood (12, 42). Patients who present with GD during childhood may experience rapid disease progression from an asymptomatic or minimally symptomatic state to an irreversible process without prompt intervention, owing to the high disease load and significant proinflammatory state. In our study, treatment with velaglucerase alfa determined a rapid decrease in Lyso-Gb1 levels within 6 months of therapy, substantial decreases in chitotriosidase activity, and normalization of spleen and liver sizes with 6 months of treatment in the majority of patients.

It is recommended that ERT is not withheld based on the identification of a genotype typically associated with neuronopathic disease (6), despite the lack of potential for a direct impact on central nervous system manifestations. It is a common belief that treating GD systemic manifestations with ERT will not impact the activity or the progressions of the GD neurological manifestations. Anecdotal evidence against this is that all neurological diseases, regardless of their etiology, are impacted by the presence of systemic inflammation, and the cause of death is mostly due to an infectious etiology, which may lead to the rapid progression of the neurological disease. For example, postmortem studies in patients with progressive multiple sclerosis unravel the role of systemic inflammation in disease progression. A newly described subset of immune cells from the systemic circulation (follicular T-helper cells) has been found to be the contributor to the primary central nervous system pathology. Although in previous studies the role of an elevated systemic inflammatory process in progressive multiple sclerosis remained unclear, the increased frequencies of Th17-cells, activated follicular T-helper cells, and B-cells that parallel neuropathologic findings suggest a pathogenic role of systemic inflammation in progressive neurologic disease (43–45). These findings support the argument against the idea that treating systemic manifestations of GD will not have any impact for GD neurological manifestations.

It is estimated that around 80 babies are born with GD each year in the USA (46). GD is not included on the US National Recommended Uniform Screening Panel (RUSP), a recommended, but not mandated, list of disorders for inclusion on the NBS panels for individual US states. However, several states have included lysosomal disorders on the RUSP in their NBS panels, including GD (47). The results from a Delphi consensus supported NBS for GD, given that earlier onset and progression of GD signs correlate with more severe disease and a high risk of morbidity (33). Nevertheless, the panel also agreed that NBS is likely to identify individuals who may not require immediate treatment (6). Importantly, all the infants identified through NBS in this study presented with splenomegaly, defying the above assumption. Patients carrying *GBA1* alleles predictive of severe disease will require a more involved therapeutic approach and management than the “wait and see” method. Additional pilot studies on the outcomes of early treatment of GD, such as this study, will be crucial in demonstrating the potential benefits of NBS for this condition (6, 33).

Cerezyme (imiglucerase) is the most widely used ERT for GD, but data in infants and children under 4 years are limited. Earlier single case reports described a 3-year-old with GD1 treated with imiglucerase, resulting in normalization of spleen size and hematological parameters after 24 months, consistent with our observed outcomes (48). In a case series (49), six infants with GD (aged 6–24 months at initiation), showed improvements in hepatosplenomegaly and hematological parameters within 6–12 months with imiglucerase, similar to our findings with velaglucerase alfa. However, infusion-related reactions were reported in two patients, unlike the absence of such events in our cohort. However, the lack of anti-drug antibody testing limits our ability to fully assess immunogenicity of velaglucerase alfa in this young age group. Another retrospective study (50) included children under 4 years treated with imiglucerase, reporting reduced organomegaly but variable bone outcomes. This current study suggests that velaglucerase alfa offers both efficacy in very young children, and a favorable safety profile.

There are limitations to our report. First, the findings are based on a small number of patients. Twenty patients were planned to be included; however, the study was closed early after almost 2 years of enrollment, owing to challenges in part attributable to the COVID-19 pandemic. Indeed, in a survey of healthcare professionals involved in the Fabry, Gaucher, and Hunter Outcome Survey registries, over 80% of respondents were concerned that the reliance on virtual consultations during the pandemic had, to some extent, resulted in delays in diagnosis of lysosomal disorders (51). The known and considerable heterogeneity of disease presentations in GD was also evident in our population because three patients developed progressive signs and symptoms consistent with GD2 after initially being considered to be eligible for the study. Second, since patients were treated in accordance with the physician treatment plan per standard clinical practice at the center, follow-up visits were based on the needs of the patient rather than a regular protocol-defined schedule, and not all outcomes were assessed at each of the visits. Finally, we acknowledge that this is an observational study without a comparator arm.

In conclusion, these preliminary findings show that in infants and young children with GD1 and GD3, velaglucerase alfa, administered mostly via home infusions, is well tolerated when initiated under 4 years of age and is associated with positive clinical outcomes.

## Data Availability

The datasets presented in this article are not readily available because privacy restrictions exist owing to the small number of study participants. Requests to access the datasets should be directed to the corresponding author.
